# Sulfonal in the Treatment of the Insane

**Published:** 1894-05

**Authors:** 


### Selections and Abstracts.

Sulfonal in the Treatment of the Insane.—Dr. John H. Scally (Maryland Hospital for the Insane) reports as follows concerning the action of sulfonal: "In treatment at this hospital, sulfonal has been used for its hypnotic effect in the stages of excitement during attacks of acute mania, mania following epilepsy, recurrent mania, chronic mania, and also in melancholia. It has not been our custom to give it regularly each day, but only at those times when, owing to the extreme restlessness and motor excitability of patients, sleep is denied them. In the management of acutely maniacal patients just admitted, when it is necessary to secure immediate rest, and, as is often the case, when the patients' very lives demand it, sulfonal has not failed in any case in which it has been used. Given in dram doses, perferably in whisky, not only has it secured from six to eight hours of sound asleep, but it has produced quite a decided amount of motor sedation, lasting from eight to twelve hours after waking. In each case sleep was obtained within one hour after administration, and in none was any bad after-effect noticed. Three of our cases, two being acute mania, and one epileptic mania, furnish evidence of the value of sulfonal as a prompt and reliable hypnotic when given in sufficiently large doses. In the first two cases both patients had been given morphia injections and other hypnotics by their family physicians with no appreciable effect. In both cases sulfonal acted promptly. In the third case sulfonal was found to act much more promptly than bromidia, paraldehyd, or morphia, all of which had been previously given."—*Ex.*

